# *CheckMyMetal* (*CMM*): validating metal-binding sites in X-ray and cryo-EM data

**DOI:** 10.1107/S2052252524007073

**Published:** 2024-08-14

**Authors:** Michal Gucwa, Vanessa Bijak, Heping Zheng, Krzysztof Murzyn, Wladek Minor

**Affiliations:** a Department of Molecular Physiology and Biological Physics University of Virginia Charlottesville22908 USA; b Department of Computational Biophysics and Bioinformatics Jagiellonian University Krakow Poland; c Doctoral School of Exact and Natural Sciences Jagiellonian University Krakow Poland; d Bioinformatics Center Hunan University College of Biology Changsha Hunan410082 People’s Republic of China; University of Auckland, New Zealand

**Keywords:** *CheckMyMetal*, *CMM*, metal-binding sites, cryo-EM, X-ray crystallography, metal ions, structural biology

## Abstract

Identifying and characterizing metal-binding sites (MBS) within macromolecular structures is imperative for elucidating their biological functions. *CheckMyMetal* (*CMM*) is a web based tool that facilitates the interactive valid­ation of MBS in structures determined through X-ray crystallography and cryo-electron microscopy (cryo-EM). Recent updates to *CMM* have significantly enhanced its capability to efficiently handle large datasets generated from cryo-EM structural analyses. In this study, we address various challenges inherent in validating MBS within both X-ray and cryo-EM structures. Specifically, we examine the difficulties associated with accurately identifying metals and modeling their coordination environments by considering the ongoing reproducibility challenges in structural biology and the critical importance of well annotated, high-quality experimental data. *CMM* employs a sophisticated framework of rules rooted in the valence bond theory for MBS validation. We explore how *CMM* validation parameters correlate with the resolution of experimentally derived structures of macromolecules and their complexes. Additionally, we showcase the practical utility of *CMM* by analyzing a representative cryo-EM structure. Through a comprehensive examination of experimental data, we demonstrate the capability of *CMM* to advance MBS characterization and identify potential instances of metal misassignment.

## Abstract 2

Recent updates to *CheckMyMetal* have significantly enhanced its capability to efficiently handle large datasets, including those generated from cryo-EM structural analyses.

## 1. Introduction

In the intricate realm of macromolecular structures, metal ions play a pivotal role, serving as essential elements for upholding structural integrity (Moura *et al.*, 2008 ▸; Zheng *et al.*, 2015 ▸) and participating as cofactors in catalytic reactions (Bowman *et al.*, 2016 ▸). Metal ions are crucial components of certain anticancer drugs (Guo *et al.*, 2023 ▸; Shabalin *et al.*, 2015 ▸) and exhibit diverse functions, exemplified by their role in various bio­logical processes, including metal signaling (Tsang *et al.*, 2021 ▸), metalloallostery (Pham & Chang, 2023 ▸), metabolism (Ackerman *et al.*, 2017 ▸), tumor progression and programmed cell death in cancer (Wang *et al.*, 2023 ▸).

Navigating the complexities of working with metal ions in macromolecular structures demands a comprehensive understanding that spans chemical, crystallographic, biological and experimental considerations. The identification and accurate modeling of metals present formidable challenges, demonstrated by the fact that 40% of macromolecular structures in the Protein Data Bank (PDB) incorporate metal ions, which are not always correctly identified and refined (Zheng, Chordia *et al.*, 2014*a* ▸). Addressing these challenges requires meticulous attention to the chemical properties of metals, their coordination chemistry and the potential impact of experimental data quality on the final structural model.

The reproducibility of biomedical research has emerged as a considerable concern (Prinz *et al.*, 2011 ▸; Begley & Ioannidis, 2015 ▸; Collins & Tabak, 2014 ▸; Baker, 2016 ▸). Structural biology has made significant progress in improving reproducibility through standardized techniques, rigorous validation processes and data-sharing initiatives like the PDB (Berman *et al.*, 2000 ▸; Burley *et al.*, 2022 ▸). The importance of these initiatives cannot be overstated, especially considering the widespread use of structural data from the PDB, with each deposit being downloaded on average around 15 000 times during 2023. Consequently, any inaccuracies in PDB entries can propagate and hinder subsequent research efforts (Zheng, Hou *et al.*, 2014 ▸). The inability to reproduce many studies often stems from incomplete or inaccurate reporting of experimental methodologies and sometimes simple negligence (Wlodawer *et al.*, 2018 ▸, 2008 ▸; Dauter *et al.*, 2014 ▸). There is a growing recognition of the necessity for tailored validation tools within each research domain (Errington, Denis *et al.*, 2021 ▸; Errington, Mathur *et al.*, 2021 ▸; Nosek & Errington, 2020 ▸).

This manuscript outlines the recent enhancements of the web tool *CheckMyMetal* (*CMM*), facilitating in-depth interactive analysis of designated metal-binding sites (MBS). *CMM* employs meticulously chosen validation parameters (Gucwa *et al.*, 2023 ▸; Zheng *et al.*, 2017 ▸; Zheng, Chordia *et al.*, 2014*b* ▸) to characterize the geometric properties of the scrutinized MBS. We investigate how the values of individual validation parameters correlate with the resolution of structures determined by X-ray crystallography (XRC) and cryo-electron microscopy (cryo-EM). Regardless of variations in resolution definitions between these methods (Dubach & Guskov, 2020 ▸; Wlodawer *et al.*, 2017 ▸), trends and dependencies in the validation parameter values of MBS can be independently determined and compared. Such analyses may potentially aid in interpreting *CMM* results for individual MBS while highlighting the complementary nature of the two most used research methods in macromolecular structure determination. The new version of *CMM* effectively handles massive datasets, allowing us to show for the first time how *CMM* effectively reduces the potential chances of metal misassignments when analyzing the results of cryo-EM experiments.

## 2. Materials and methods

### 2.1. Enhanced functionality in *CMM*

*CMM* continuously evolves to address the unique aspects of working with structural data from XRC and cryo-EM. Recent updates address the graphical user interface and the backend algorithms responsible for evaluating MBS.

The current version of *CMM* incorporates the Python Django framework [Django Software Foundation (2019), https://djangoproject.com] to facilitate user interaction and presentation of MBS analysis results. This framework offers many possibilities for further enhancements in application performance, convenience and user experience. The latest release includes a long-awaited feature for handling electron density maps: both 2*F*_o_ − *F*_c_ and *F*_o_ − *F*_c_ maps for XRC and electrostatic potential maps from cryo-EM (in MRC format). These maps are efficiently visualized using the *NGL Viewer* (Rose & Hildebrand, 2015 ▸; Rose *et al.*, 2018 ▸). Additionally, a new ‘MODEL’ tab in the workspace panel enables on-the-fly refinement of MBS in XRC structures (Vagin *et al.*, 2004 ▸), if structure factors are available. *CMM* is capable of handling large structures, as models can now be uploaded in either legacy PDB or PDBx/mmCIF formats.

On the backend side, *CMM* applies a set of criteria (Gucwa *et al.*, 2023 ▸) to evaluate alternative metals in a given binding site. These criteria are crucial for discriminating between alternative metals. Each of the validation parameters, such as ATOMIC CONTACTS, GEOMETRY and VALENCE can be labeled as DUBIOUS, BORDERLINE or ACCEPTABLE. An efficient method for evaluating atomic contacts of the neighboring metals in binding sites has been implemented to accommodate the increased complexity of contemporarily deposited structures. This involved setting a distance cutoff of 4 Å to limit interactions and skipping intra-molecular interactions primarily composed of covalent and hydrogen bonds unrelated to metal coordination. Furthermore, consideration of non-crystallographic symmetry has been introduced to speed up MBS validation. Finally, calculations of validation parameters are now streamlined and simultaneously performed for all metals considered (Na^+^, Mg^2+^, K^+^, Ca^2+^, Mn^2+^, Fe^3+^, Co^2+^, Ni^2+^, Cu^2+^ and Zn^2+^).

### 2.2. Data collection and analysis

The PDBj’s ‘*Mine 2 RDB*’ (Kinjo *et al.*, 2017 ▸, 2018 ▸; Bekker *et al.*, 2022 ▸) was utilized in the analyses described in this paper. The molecular weights of the macromolecular structures were determined by summing all entities.formula_weight fields. In rare cases, this value may be significantly overestimated. For instance, in the case of structure 6x6l, despite the long-sequence formula totaling approximately 4.7 MDa, only a small portion of amino acids, totaling 0.6 MDa, were found within the structure. Analyses described here focus solely on HETATM fields in PDB deposits with specific residue names: NA, MG, K, CA, MN, FE, CO, NI, CU and ZN. Thus, they do not include, for example, metal ions from iron–sulfur clusters (residue name: FES) or chloro­phyll with Mg^2+^ ions (residue name: CLA). *PyMOL* (The PyMOL Molecular Graphics System; Schrödinger, LLC) was employed to create figures illustrating structures of macromolecules and MBS. The plots were created with *Matplotlib* (Hunter, 2007 ▸).

## 3. Results and discussion

Structural biology results depend significantly on the choice of experimental techniques and even on the choice of a particular experimental facility (Grabowski *et al.*, 2021 ▸). X-ray protein crystallography and cryo-EM are two pivotal method­ologies, each with distinct advantages and challenges. Aside from ensuring the quality of the data obtained from XRC or cryo-EM experiments, other factors significantly influence the final quality of MBS, such as sample preparation and effective management of the multitude of MBS during the experiment, modeling process and model refinement. A good understanding of the nature of MBS and resolution capabilities across XRC and cryo-EM sets the stage for exploring factors that may influence the modeling of MBS. In the case study presented, researchers witness firsthand how factors such as resolution quality and the complexity of MBS influence the modeling, which may be easily overlooked. The careful use of *CMM* allows for the detection of previous misassignments, leading to the discovery of new and intriguing insights in present research.

### 3.1. Macromolecular size complementarity of XRC and cryo-EM

**Figure 1:**
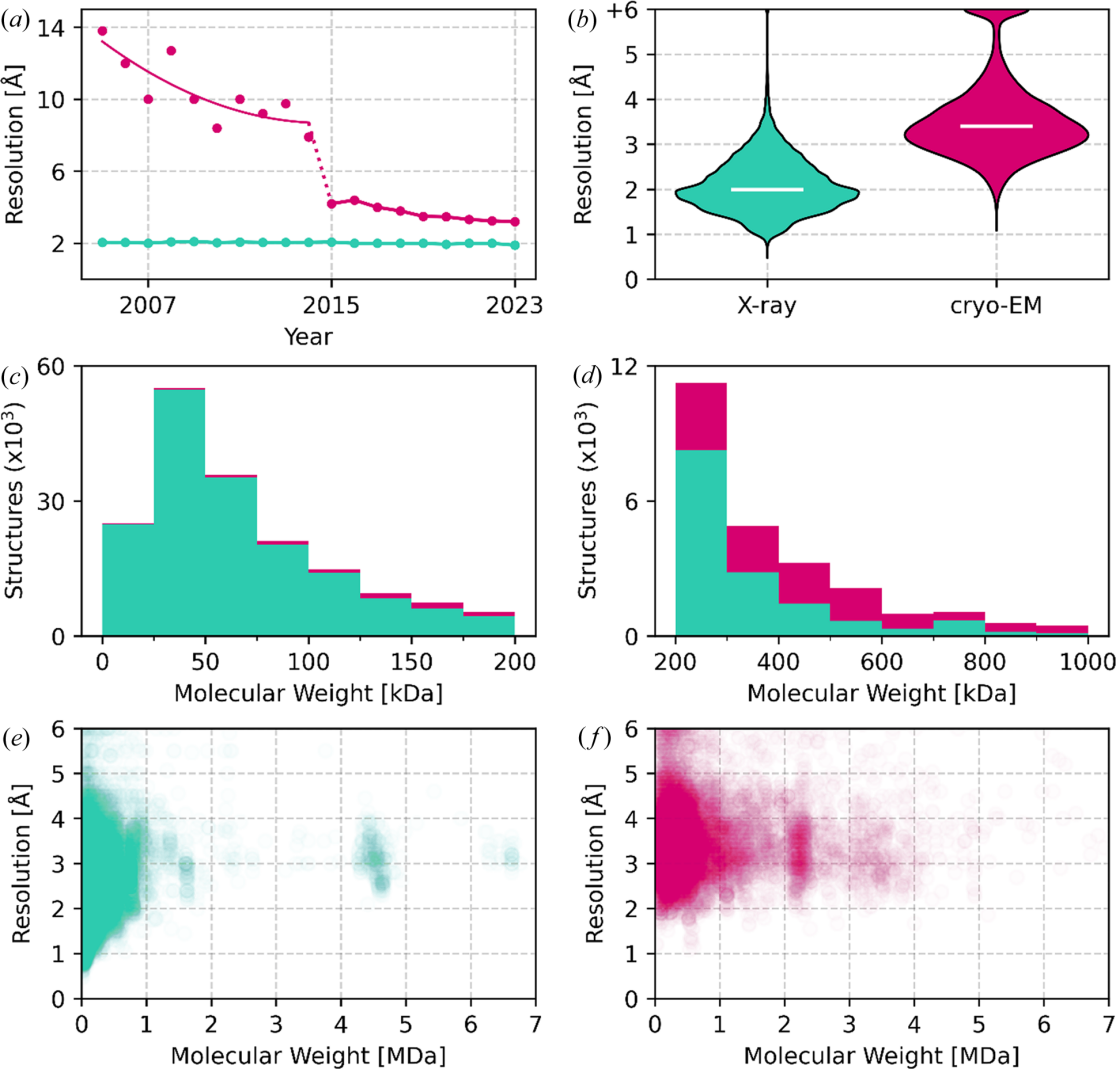
Distribution of resolution and molecular weight across XRC (teal) and cryo-EM (magenta) macromolecular structures. (*a*) Median resolution in PDB deposits in individual years. (*b*) Violin plots for resolution of macromolecular structures in 2015–2023. White lines represent the median resolution of 3.4 Å for cryo-EM and 2.0 Å for XRC. (*c*) and (*d*) Distribution of molecular weight in PDB structures, presented as a stacked histogram. (*e*) and (*f*) Scatter plots for resolution versus molecular weight. One data point corresponds to a single PDB deposit. With its opacity set to 0.01, it takes 100 PDB deposits to make a plot dot fully opaque.

XRC allows the collection of high-resolution data, as shown in Fig. 1 ▸(*b*), with the highest resolution achieved being 0.48 Å for the high-potential iron–sulfur protein (5d8v; Hirano *et al.*, 2016 ▸). However, obtaining diffraction-quality crystals poses significant challenges, particularly for large protein complexes (Mueller *et al.*, 2007 ▸) and/or membrane proteins (Carpenter *et al.*, 2008 ▸). Consequently, successful applications of XRC to study biological structures exceeding 800 kDa are rare, accounting for only 0.5% of X-ray structures [Figs. 1 ▸(*c*), 1 ▸(*d*) and 1 ▸(*e*)]. A number of mission-impossible projects, like the work of Yonath, Ramakrishnan and Steitz (Harms *et al.*, 2001 ▸; Murphy & Ramakrishnan, 2004 ▸; Ban *et al.*, 2000 ▸) have been recognized by Nobel committees. The two high-molecular-weight regions presented in Fig. 1 ▸(*e*) (∼4.5 MDa, ∼6.6 MDa) are also ribosome-related work (Melnikov *et al.*, 2016 ▸; Batool *et al.*, 2020 ▸). Due to the inclusion of two ribosomes within an asymmetric unit in the XRC structures, these two regions corresponding to the 70S and 80S ribosomes, correspond to regions ∼2.2 and ∼3.2 MDa in Fig. 1 ▸(*f*), respectively, as cryo-EM structures typically contain a single ribosome complex per deposit.

Although cryo-EM traditionally lagged behind XRC in resolution [Fig. 1 ▸(*a*)], 2015 marked a milestone, representing a leap forward in its development [Fig. 1 ▸(*a*)] (Cheng, 2015 ▸). The highest-resolution cryo-EM structure achieved so far is the 1.15 Å structure of human apoferritin (7a6a; Yip *et al.*, 2020 ▸). As a result, cryo-EM has become increasingly competitive in the determination of sophisticated details of biomolecular architectures, particularly for larger protein complexes and assemblies, which are very difficult to crystallize [Figs. 1 ▸(*d*) and 1 ▸(*f*)].

### 3.2. Cognitive overload in validation of MBS

The importance of resolution and accuracy in MBS structures cannot be emphasized enough, particularly in rational drug design (Bijak *et al.*, 2023 ▸). Although resolution is described differently for structures determined in XRC and cryo-EM, it usually correlates well with the overall accuracy of experimental data in both methodologies (Wlodawer *et al.*, 2017 ▸).

**Figure 2:**
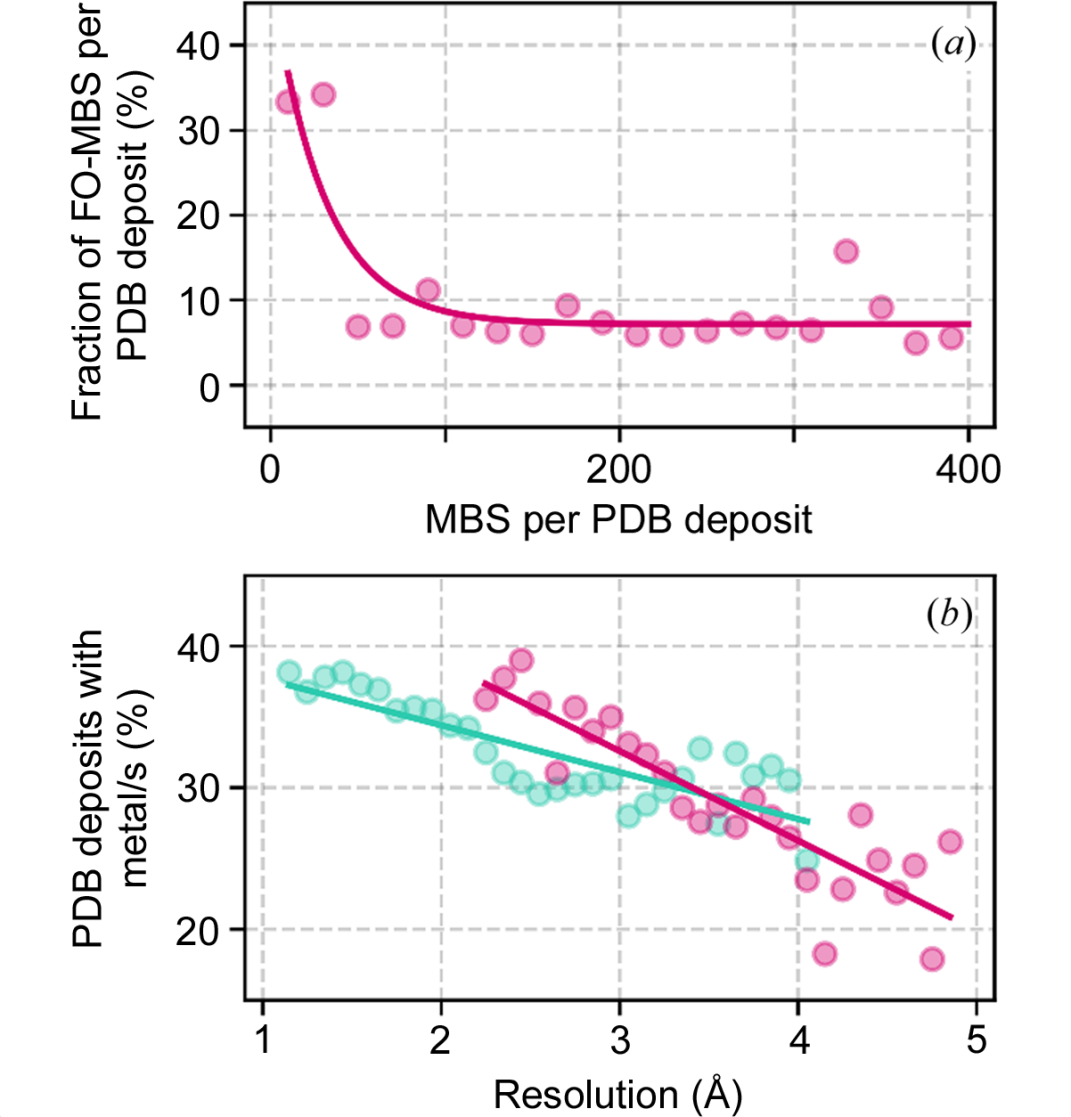
Cognitive overload in MBS identification in XRC (teal) and cryo-EM (magenta) structures. (*a*) Percentage of MBS with a filled coordination sphere (fully occupied MBS) as a function of the number of MBS in a cryo-EM structure. A single data point represents the median value of all cryo-EM PDB deposits within consecutive intervals of 20 MBS. (*b*) Percentage of structures with at least one metal-binding site. A single data point represents a percentage value in 0.1 Å intervals. In both plots, the solid lines are drawn as a guide to the eye.

MBS typically consist of a metal ion coordinated by amino acid residues and water molecules that complete the first coordination sphere. As shown in Fig. 2 ▸(*a*), the fraction of PDB deposits containing fully occupied MBS decreases when the number of MBS exceeds 40, highlighting a constraint that researchers encounter when validating numerous MBS in a structure. This cognitive overload arises when the ability to process information is overwhelmed by its volume or complexity, resulting in difficulties in decision making or comprehension. Our analysis underscores the challenge researchers face, as a higher number of MBS seems to deter scientists from dedicating significant attention to their modeling (Fig. S1 of the supporting information). Recognizing this issue, *CMM* offers assistance by identifying which MBS require further attention to prioritize modeling efforts.

Fig. 2 ▸(*b*) shows that, for XRC and cryo-EM, the number of MBS decreases significantly with decreasing resolution. There is a higher likelihood of overlooking MBS at worse resolutions, leading to their exclusion from the structural refinement process due to inadequate data accuracy. Therefore, high-resolution data are essential for the correct identification of all MBS.

### 3.3. Dependence of *CMM* validation parameters on structure resolution

Obtaining high-resolution macromolecular structures containing metal ions presents several significant challenges, whether XRC or cryo-EM. In XRC, difficulties often arise due to the radiation sensitivity of metals, leading to radiation damage and subsequent reduction in data quality. MBS may also exhibit disorder or varied occupancies, rendering structure determination and refinement relatively difficult. Additionally, obtaining well ordered protein crystals containing metal ions can be challenging due to their inherent flexibility or the presence of solvent-accessible MBS. Similarly, in cryo-EM, metal ions can contribute to increased specimen heterogeneity or cause beam-induced motion, affecting image quality and limiting achievable resolution. Furthermore, metal-induced phase contrast can obscure fine structural details, complicating the interpretation of density maps.

We analyzed MBS in structures determined in XRC and cryo-EM using a subset of *CMM* validation parameters: VALENCE, nVECSUM, gRMSD and VACANCY. Each of these parameters is classified as DUBIOUS, BORDERLINE and ACCEPTABLE according to the criteria used in *CMM* (Gucwa *et al.*, 2023 ▸) and determined as a function of structure resolution. From the analysis of profiles obtained for XRC structures, it follows that nVECSUM values, even in the range of high resolutions (<2 Å), are acceptable for at most 50% of the validated MBS. The remaining validation parameters in this range differentiate the analyzed MBS to a lesser extent. For low-resolution structures (>2.7 Å), the accuracy of modeling MBS is already low and rapidly decreases with deteriorating resolution. The same observations can be made when analyzing profiles for cryo-EM, with the difference that, for comparable resolutions, *CMM* validation parameters in cryo-EM structures consistently take lower values than in the case of XRC. This is not surprising, as it is much better and easier to model an MBS with good-quality, high-resolution data [see Fig. S2(*a*)] rather than poor-quality, low-resolution data [see Fig. S2(*b*)]. Note that the identification of metals is more difficult using cryo-EM than XRC (Fig. S3), because the anomalous and difference density maps are unavailable (Fig. S3).

**Figure 3:**
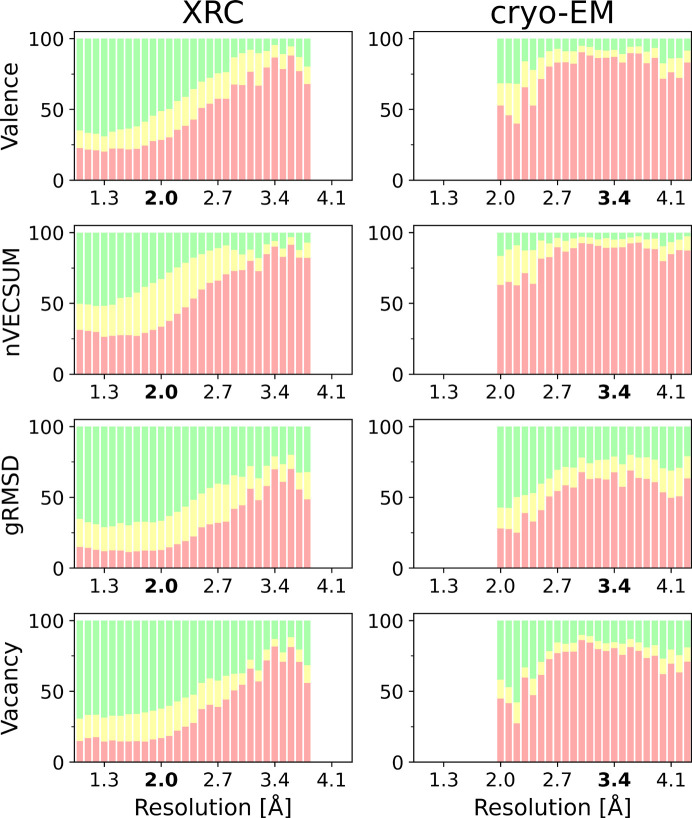
Stacked bar plot analysis of the *CMM* validation parameters for XRC (left) and cryo-EM (right). Green, yellow and red bars correspond to the specifically labeled categories ACCEPTABLE, BORDERLINE and DUBIOUS, respectively.

A significant aspect in validating MBS is the relationship between structure resolution and the completeness of the coordination sphere. In metalloproteins, vacant positions within the first coordination sphere of a metal ion are frequently observed, particularly on the protein surface. Here [Fig. S2(*a*)], the metal ion forms coordination bonds with surrounding amino acid residues and water molecules. High-resolution structures typically reveal distinct electron density peaks, facilitating the identification of all coordinating atoms and overall MBS geometry. Conversely, low-resolution structures pose challenges as they usually show just a single poorly resolved electron density blob which may not even encompass the entire MBS, making the assignment of water oxygen atoms in the first coordination sphere extremely difficult. Consequently, researchers may opt not to assign full occupancy, leaving some coordination positions vacant [Fig. S2(*b*)]. This scenario appears to be confirmed by the dependence of the acceptable values of the VACANCY parameter on resolution in XRC structures (Fig. 3 ▸, Vacancy), where a plateau is observed followed by a linear decrease in the percentage of acceptable values for lower resolutions.

### 3.4. A *CMM* case study: 70S ribosome with tRNAs

This section describes an example scenario of using *CMM* to validate MBS in the cryo-EM structure 8b0x (Fromm *et al.*, 2023 ▸). This cryo-EM structure determined at 1.55 Å resolution is among 14 cryo-EM structures at a resolution of 1.6 Å or better [Fig. 1 ▸(*b*)].

The 8b0x structure contains 530 metal ions, comprising 361 Mg^2+^ ions, 168 K^+^ ions and 1 Zn^2+^ ion. The authors implemented a strategic approach to identify K^+^ and Mg^2+^ ions using previously determined structures 6qnr (Rozov *et al.*, 2019 ▸) and 7k00 (Watson *et al.*, 2020 ▸), respectively. This methodology saves time as identifying hundreds of MBS *de novo* is very laborious. We utilize *CMM* to validate MBS in the 8b0x structure, aiming not only to test the advantages and disadvantages of the target/template approach, but also to emphasize the critical role of highly accurate experimental data availability. By showcasing the reusability of such data, we underscore the broader significance of ensuring the quality and reliability of experimental structures, thereby facilitating robust structural analyses and advancing our understanding of macromolecular interactions.

Our assessment revealed that the assignments of K^+^ ions in the 8b0x structure were generally accurate. However, we also identified 27 instances where the assignment of Mg^2+^ ions did not correlate well with the cryo-EM map and *CMM* score (Table S1 of the supporting information). Consequently, while the target/template approach provides valuable insights, it underscores the importance of caution when extending MBS assignments from other structures without thoroughly analyzing the experimental data.

**Figure 4:**
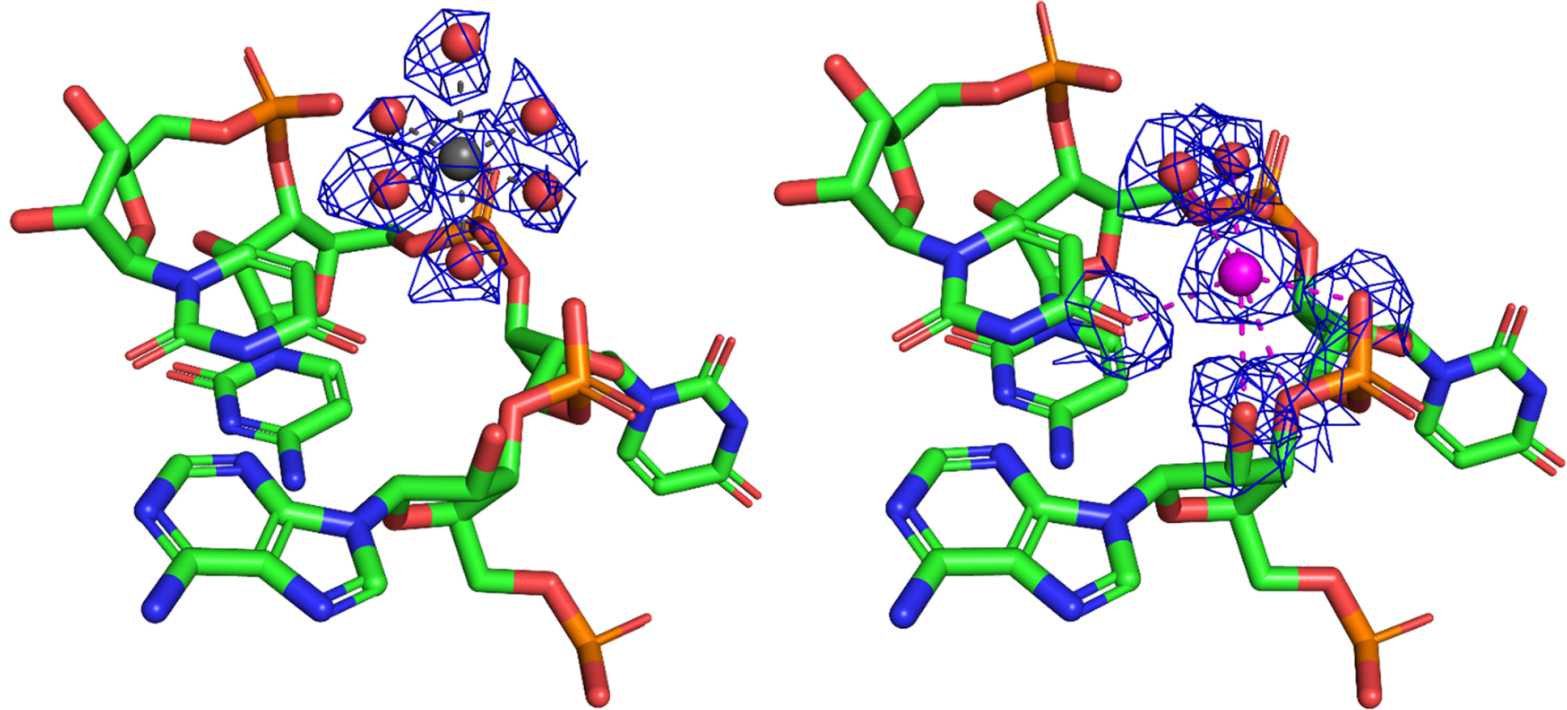
Analysis of MBS in cryo-EM structures. This figure illustrates the model with superimposed density maps for a specific metal-binding site. (*a*) Structure 7k00 (MBS ID: a:6163), showcasing a gray Mg^2+^ ion surrounded by coordinating water molecules, with modeled octahedral geometry and distances (2.25 Å) suggesting Mg^2+^ ion coordination. (*b*) Structure 8b0x (MBS ID: a:3203), depicting a purple K^+^ ion coordinated by both water molecules and rRNA residues within the first coordination sphere. The structural differences between the two representations indicate distinct metal ion coordination environments, with the K^+^ ion in 8b0x exhibiting a larger coordination distance (2.90 Å) compared with the Mg^2+^ ion in 7k00.

On further investigation, we carefully compared the 8b0x with 6qnr and 7k00 structures. In sample preparation, the 7k00 structure exclusively used only Mg^2+^ ion buffers, whereas 6qnr and 8b0x applied both Mg^2+^ and K^+^. The 7k00 structure determined at a resolution of 1.98 Å enabled the authors to identify Mg^2+^ based on octahedral geometry in the map. The XRC structure 6qnr is characterized by a much worse resolution of 3.10 Å, insufficient to observe the octahedral geometry clearly. The authors of the 6qnr structure relied entirely on anomalous signals to identify the positions of K^+^ ions and subsequently assigned Mg^2+^ ions to all density blobs that did not have an anomalous signal in the structure. However, in the 8b0x structure, with 27 MBS incorrectly identified as Mg^2+^ ions, there were 8 MBS that were found in 6qnr and 7k00 identified as Mg^2+^. Our analysis indicates that these 8 MBS in 8b0x are in fact K^+^ (Table 1 ▸, Fig. 4 ▸). One possible explanation is that 8b0x is not exactly the same as 6qnr because the MBS have slightly different conformations due to the different number of bound tRNA molecules. It was shown previously that binding different metal ions in the same MBS can be associated with a change in conformation (Declercq *et al.*, 1991 ▸), and the binding of ligands can be associated with conformational changes (Wu *et al.*, 2023 ▸). This observation underscores the importance of adequately considering the most probable metal ions for given MBS based on experimental data to avoid hindering the analysis of mechanisms in action.

In the 8b0x structure, we observed a mixture of K^+^ and Mg^2+^ ions (see Table 1 ▸, bold). This phenomenon may stem from the heterogeneous nature of cryo-EM imaging, where individual ribosomes in the sample may bind either Mg^2+^ or K^+^ ions, resulting in a composite density map showing evidence for both ions within the same MBS. Such interchangeability in metal binding shows the importance of providing detailed information about sample preparation. Since there is no mechanism to include sample preparation details in PDB deposits, this information is only available when the structure is published. Over 1200 cryo-EM deposits do not have an associated publication.

## 4. Conclusions

The importance of accurately identifying and characterizing MBS in macromolecular structures for understanding their biological functions has been emphasized in this paper. Introducing an upgraded version of *CMM* offers a valuable tool for systematically analyzing MBS in XRC and cryo-EM data, addressing challenges in metal identification and modeling. Through practical examples of the 8b0x structure of 70S ribosome and comparative analyses of output data from the *CMM* validation server, we have demonstrated the power of *CMM* in enhancing the quality and reproducibility of structural biology research. Furthermore, improvements in the *CMM* algorithms have facilitated faster and more accurate analysis, particularly for cryo-EM structures. It is evident that the systematic validation of MBS provided by *CMM* contributes to advancing our understanding of metal ion function in biological macromolecules and holds promise for future research endeavors in structural biology.

## Supplementary Material

Supporting figures and table. DOI: 10.1107/S2052252524007073/be5298sup1.pdf

## Figures and Tables

**Table 1:** Comparison of MBS in structures 6qnr, 7k00 and 8b0x The 7k00 structure served as the template for identifying Mg^2+^ MBS in the target structure 8b0x. Each row corresponds to a unique metal-binding site of the ribosome found in both PDB structures, specified by the chain, residue ID and modeled metal ion. The ‘*CMM*’ column presents the metal ion proposed by *CMM* for each metal-binding site, evaluated based on the inspection of the density map and *CMM* score. For the first three 8b0x MBS in the table (bold), the analysis indicates the coexistence of K^+^ and Mg^2+^ ions.

PDB entry 6qnr: three tRNAs in P, E and A sites	PDB entry: 7k00 (Mg^2+^ template) two tRNAs in P and A sites	PDB entry 8b0x (target) one tRNA in the P site	
MBS ID	MBS ID	MBS ID	*CMM*
13:1666 Mg^2+^	A:1668 Mg^2+^	**A:1682 Mg^2+^**	**K^+^**
1H:3446 Mg^2+^	a:6207 Mg^2+^	**a:3029 Mg^2+^**	**K^+^**
1H:3421 Mg^2+^	a:6178 Mg^2+^	**a:3005 Mg^2+^**	**K^+^**
13:1658 Mg^2+^	A:1644 Mg^2+^	A:1609 Mg^2+^	K^+^
—	A:1631 Mg^2+^	A:1714 Mg^2+^	K^+^
1H:3257 Mg^2^	a:6170 Mg^2+^	a:3032 Mg^2+^	K^+^
1H:3433 Mg^2+^	a:6143 Mg^2+^	a:3162 Mg^2+^	K^+^
1H:3357 Mg^2^	a:6163 Mg^2+^	a:3203 Mg^2+^	K^+^

## Data Availability

The authors confirm that the data supporting the findings of this study are available within the article and its supporting information.
