# Supplementary material for: CheckMyMetal (CMM): validating metal-binding sites in X-ray and cryo-EM data
Source: IUCrJ. 2024 Aug 14;11(Pt 5):871–7. doi: 10.1107/S2052252524007073 (PMC11364027; doi:10.1107/S2052252524007073)
Supplement: Supplementary file 1 [file m-11-00871-sup1.pdf]

IUCrJ

Volume 11 (2024)

Supporting information for article:

CheckMyMetal (*CMM*): validating metal-binding sites in X-ray and cryo-EM data

Michal Gucwa, Vanessa Bijak, Heping Zheng, Krzysztof Murzyn and Wladek Minor

Figure S1. A) An MBS (PDB ID: 8b0X, chain a, residue ID: 3072) featuring a magnesium ion coordinated by only three oxygen atoms. Despite the cryo-EM map providing adequate details, the remaining three coordinating positions are unoccupied in 8B0X. B) The octahedral MBS exhibits complete occupancy, with water oxygen atoms occupying the vacant positions in the cryo-EM map, thereby achieving full occupancy for this MBS.

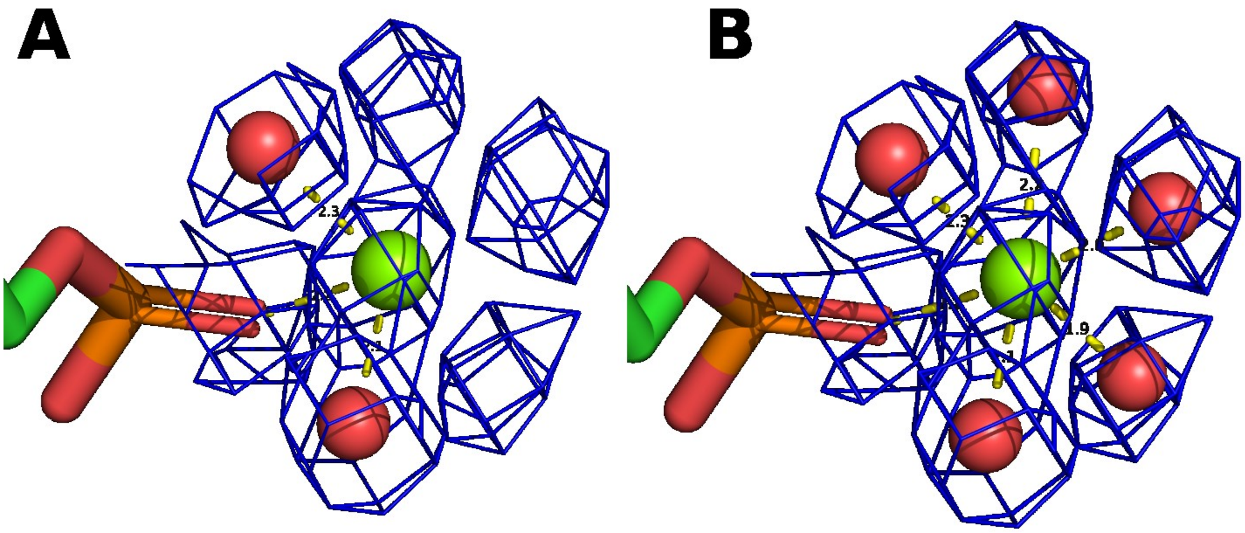

Figure S2. Example MBS observed at various resolutions (with the relative electron density contour scale set to 1). A) Structure 4E3Y determined at 1.0Å: The well-defined details of the electron density map enable the identification of individual water oxygen atoms as distinct blobs, facilitating the accurate determination of coordination geometry (in this case, octahedral). B) Structure 6CUX determined at 4.1Å: The poorly resolved electron density blob complicates the modeling of the MBS and leads to ambiguity in determining the coordination geometry.

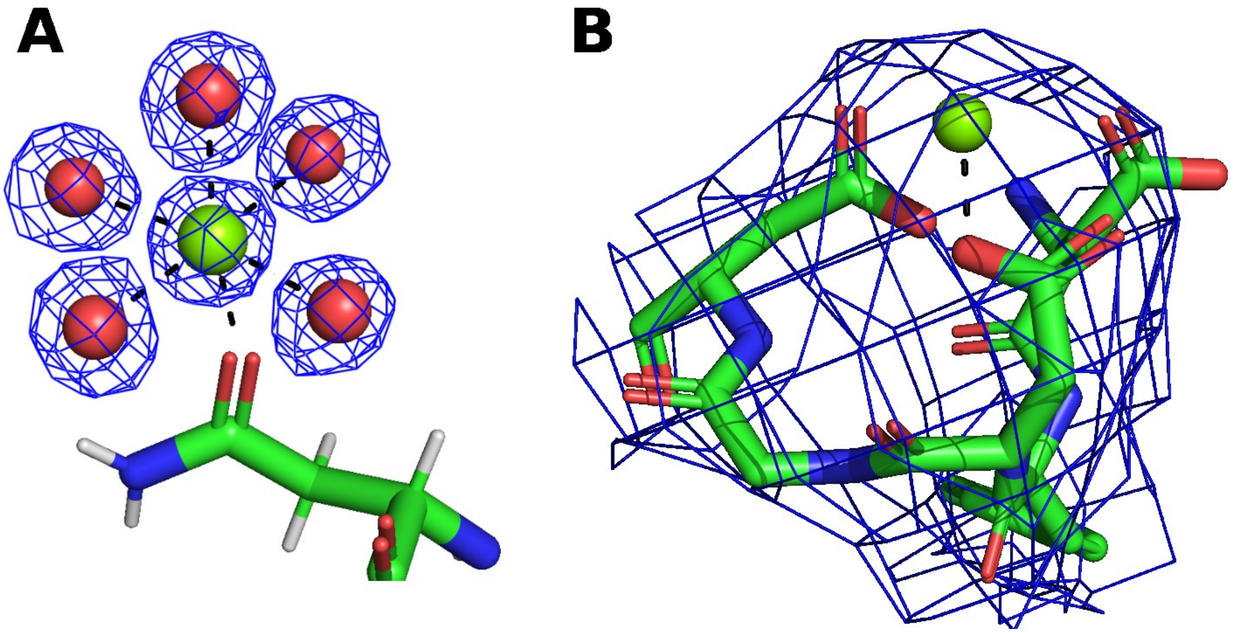

Figure S3. Utilizing difference maps for metal ion identification in structure 3C9U (XRC). In contrast to cryo-EM, XRC benefits from electron density difference maps which make easier MBS validation. A) MBS featuring a Mg^{2+} reveals a prominent positive blob in the difference map (the relative electron density contour scale set to 3.5). B) After refinement with a Ca^{2+}, the same MBS exhibits much smaller blobs in the difference map. This metal ion was selected for refinement due to its presence in the crystallization conditions (calcium acetate). Additionally, the CMM assigned a score of 6 points to Ca^{2+} compared to just 4 points for Mg^{2+}. The VALENCE parameter emerged here as a distinguishing factor, indicating that the metal-atomic contact distances are too long for Mg^{2+}, making Ca^{2+} a much better fit for this MBS.

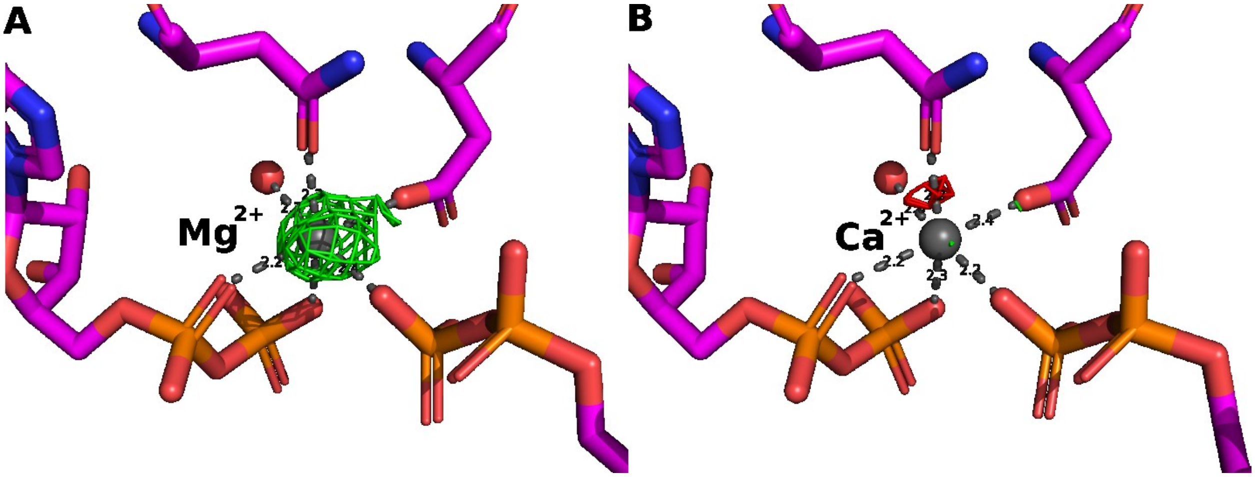

Table S1. Assessment of metal ions in structure 8B0X. Details for individual MBS with Mg^{2+} in the 8B0X deposit that are likely misidentified. Each row corresponds to a unique MBS denoted by the chain and residue IDs. The CMM scores for K+ and Mg2+ ions are provided in each case.

Chain ID:Residue ID	CMM Score for K^+	CMM Score for Mg^{2+}
A:1609	6	4
A:1636	5	2
A:1637	4	1
A:1640	4	3
A:1641	4	2
A:1682	6	4
A:1714	6	4
A:3005	5	4
A:3029	5	4
A:3031	6	4
A:3032	5	2
A:3042	2	1
A:3044	5	0
A:3045	4	2
A:3051	5	4
A:3052	4	1
A:3053	6	4
A:3054	5	2
A:3055	6	2
A:3068	5	4
A:3069	3	2
A:3070	6	4
A:3162	3	3
A:3203	6	4
A:3358	4	2
A:3365	5	2
A:3366	5	2
